# Diagnostic value of ultrasonographic measurement of peritoneal, mesenteric and intestinal wall thickness in children with peritoneal dialysis-related peritonitis: a preliminary clinical study

**DOI:** 10.1186/s12880-026-02518-6

**Published:** 2026-06-18

**Authors:** Lujie Dong, Xiaojing Lin, Shu Zhu, Yaping Liu, Yu Zhang, Hongchang Luo

**Affiliations:** 1 Department of Medical Ultrasound, Tongji Hospital, Tongji Medical College, Huazhong University of Science and Technology, Wuhan, Hubei China; 2 Department of Pediatric Nephrology, Tongji Hospital, Tongji Medical College, Huazhong University of Science and Technology, Wuhan, Hubei China

**Keywords:** Ultrasound, Peritoneal dialysis, Intestinal wall thickness, Peritonitis, Children

## Abstract

**Background:**

Peritonitis is a common serious complication of peritoneal dialysis. Although peritoneal thickening is commonly observed in long-term peritoneal dialysis patients, there have been relatively few studies on mesenteric and intestinal wall alterations through ultrasonography, particularly in pediatric patients. We aim to determine the value of ultrasonographic measurement in children with peritoneal dialysis-related peritonitis.

**Methods:**

We recruited two groups of pediatric patients, of whom 40 had peritonitis (study group) and 42 had no peritonitis (control group). We measured peritoneal, mesenteric and intestinal wall thickness by trans-abdominal ultrasonography. To identify risk factors and develop a predictive nomogram for peritonitis, we employed multivariate logistic regression analysis. Subsequently, the performance of the nomogram model was assessed through the ROC curve, calibration curve, and decision curve analysis (DCA).

**Results:**

The ultrasonographic measurements of the peritoneal thickness, mesenteric thickness, jejunal wall and ileal wall thickness in peritonitis group were significantly thicker than the non-peritonitis group. The peritonitis group had significantly higher NLR (neutrophil-to-lymphocyte ratio) and significantly lower albumin and uric acid levels than the non-peritonitis group. Multivariate Logistic analysis showed that dialysis duration, mesenteric thickness, jejunal wall thickness and NLR were potential associated factors. ROC curve showed that the integrated predictive model exhibited superior diagnostic accuracy (AUC = 0.873) over that of any single predictive parameter (*P* < 0.05). The nomogram model showed good calibration and clinical benefit.

**Conclusions:**

High-frequency ultrasound effectively supports the diagnosis of peritoneal dialysis-related peritonitis. The integrated predictive model based on ultrasonographic measurements is effective for predicting the recurrent PD-related peritonitis in children and offering practical ultrasonographic criteria.

## Introduction

Chronic kidney disease(CKD) is a progressive condition that affects approximately 10% of the global population, roughly 800 million people worldwide, and over 82 million individuals in China had been diagnosed by the end of 2022 [1, 2]. In the pediatric age group, CKD is rare with reported incidence of 4–14 cases per million and a prevalence of 18–100 cases per million of the age-related population [3]. Peritoneal dialysis (PD) is the preferred kidney support therapy for children with kidney failure due to its wide applicability and good compatibility with daily life, and it is also the predominant maintenance dialysis modality in this age group, serving as an essential bridge until successful renal transplantation can occur [4].

Peritonitis is one of the main complications of PD, which results in considerable morbidity and mortality and significantly limits the use of dialysis modality. Available evidence demonstrates that long-term PD, together with repeated episodes of peritonitis, lead to increased peritoneal thickness (most focusing on the parietal peritoneum) [5–8]. Besides the parietal peritoneum, the visceral peritoneum (including mesenteric and intestinal wall) also plays a significant role in the PD process, constituting a significantly larger proportion of the surface are involved. Moreover, it is equally susceptible to changes during the life-long course of PD. There is relatively little research on the relationship between peritonitis and peritoneal, mesenteric and intestinal wall thickness, especially in children. Thus, we attempted to investigate the relationship between changes in peritoneal, mesenteric and intestinal wall thickness with PD-related peritonitis.

Previous studies that investigated the morphology and functions of the peritoneum have used histological and histomorphometrical methods through biopsy [5, 6, 8], which is an invasive method. Ultrasound has demonstrated unique advantages as a noninvasive, radiation-free, convenient, and highly repeatable imaging technology in the diagnosis and management of peritonitis in patients undergoing PD. ISPD recommended US as a gradual diagnostic examination [9, 10]. A few of studies have established that ultrasonography (USG) of the parietal peritoneum is a reliable and reproducible fashion [7, 11, 12]. However, there is currently no literature reporting the USG structural properties of the visceral peritoneum resulting from PD-related peritonitis. Our study aims to employ ultrasound measurements to assess the peritoneal, mesenteric and intestinal wall thickness in children undergoing PD, with the objective of further exploring their diagnostic value in PD-related peritonitis.

## Methods

### Subjects

This single-center, retrospective, observational and comparative study was conducted on 82 children (43 females, 39 males) with kidney failure undergoing PD between April 2021 and March 2025, at the Department of Pediatric Nephrology, Tongji Children’s Hospital, China. Study participants were grouped based on complication of peritonitis into peritonitis group and non-peritonitis group. The peritonitis group included patients with either a single peritonitis attack or recurrent episodes, and all episodes were taken into account in the final analysis. The ages of patients in both groups ranged between 0 and 14 years.

Our inclusion criteria were children who have undergone PD treatment for at least 3 months. Our exclusion criteria were children who had a history of abdominal surgery or malignancy, intestinal diseases, and other infections that could cause acute thickening of the peritoneum or intestinal wall. Children with amyloidosis were excluded due to the potential thickening of the peritoneal membrane associated with this condition. The sex, height, weight and body mass index (BMI) were measured and recorded for every child. The values were computed. Dialysis duration and laboratory data were obtained from patient records. All laboratory measurements were obtained at least 4 weeks after peritonitis resolution during stable PD follow-up.

### Ultrasonographic measurements

The USG measurements were implemented under strictly standardized conditions. All participants fasted for 4–6 h before scanning to minimize intestinal gas artifact. No sedation was administered, and all children completed the examination with the help of their guardians. A standardized dialysate volume of 40 mL/kg was instilled into the peritoneal cavity for every patient, followed by a 30-minute dwell time prior to ultrasound acquisition. This unified dialysate volume and dwell time standardized intra-abdominal pressure and bowel distension status, optimized the visualization of target structures, and prevented measurement variations caused by inconsistent intraperitoneal conditions. All measurements were performed in the supine position using a 12 MHz linear array probe (GE Logic E9, Wauwatosa, United States) at fixed scanning windows. The fixed scanning positions for USG measurements were determined according to the study by Faller et al. [11]. Horizontal sections were acquired along the midsternal line in the left upper quadrant, right upper quadrant, left lower quadrant and right lower quadrant.

#### The parietal peritoneum

**Fig. 1:**
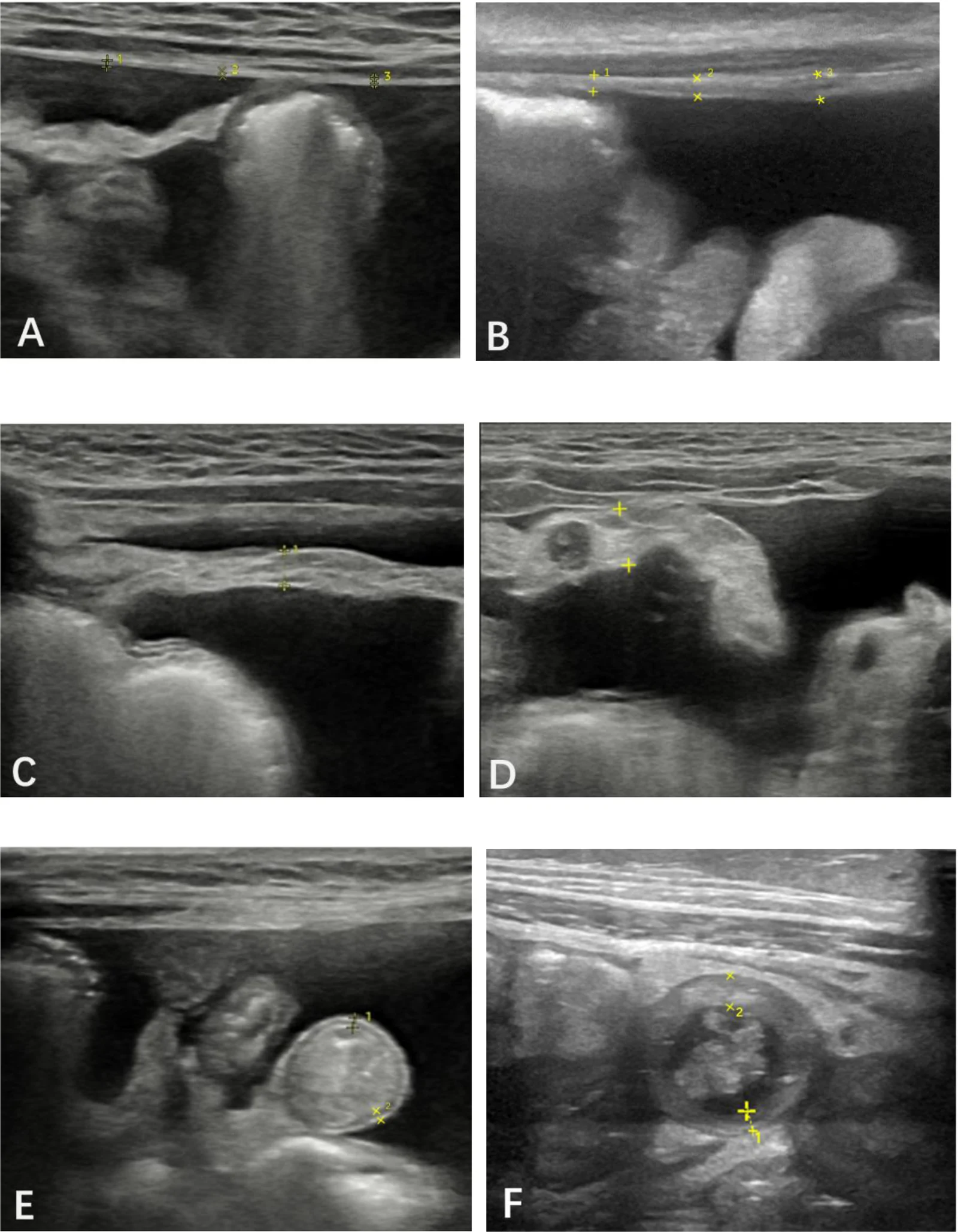
The USG appearance of peritoneum, mesentery and intestinal wall in a 5-year-old child with no peritonitis (**A**, **C**, **E**) and in a 7-year-old child with 4 times peritonitis (**B**, **D**, **F**). The parietal peritoneal zone thickness was measured in 3 distinct sites(**AB**). The measurements of intestinal wall were repeated with the cross-sectional angle > 90° between two measurement axes (**EF)**

We measured parietal peritoneum thickness at lower quadrant of abdomen without the port of Tenckhoff catheter. The primary focus of trans-abdominal USG examination was the sub mesothelial compact collagen zone, characterized by a distinct, quantifiable hyperechoic layer (Fig. 1, AB). We took parietal peritoneum thickness measurements from the lower abdominal quadrant based on existing literature reported that this region had both greater absolute thickness (mean ± SD: 1.07 ± 0.85 mm vs. upper abdomen’s 0.59 ± 0.40 mm) and wider physiological fluctuation ranges (range: 0.48–1.07 mm vs. 0.27–0.59 mm in other quadrants) has been observed [7, 11].

#### The mesentery and intestinal wall

Due to the absence of fixed anatomical landmarks, accurately distinguishing specific segments of the jejunum and ileum remains challenging. The proximal jejunum is typically observed in the left upper abdominal quadrant, characterized by prominent plicae circulares and active peristalsis. The terminal ileum can be observed in the right lower quadrant, where mucosal folds gradually diminish and ultimately disappear, accompanied by reduced peristaltic activity. When measuring the terminal ileum and mesentery, the probe was initially positioned along the longitudinal axis of the ascending colon and then advanced distally to the site where the bowel, ileum, and appendix converge in the right lower quadrant. Then, the terminal ileum and its mesentery were detected. The measurements of terminal ileum mesentery were performed in the midline on longitudinal sections (Fig. 1, CD). On the selected cross-section of the intestinal segment, calipers were placed perpendicular to the wall. Measurements were repeated with the cross-sectional angle more than 90° between two measurement axes [13] (Fig. 1, EF).

All ultrasound scans were performed uniformly at 4 weeks after the latest peritonitis episode. All recordings and measurements were made at the maximal enlargement of the still photographs. All values were expressed as the mean of three measurements± standard deviation in a unit of mm. All USG measurements were made by a single sonographer who was blinded to the peritonitis history to reduce the examiner-related error. Intra-observer reproducibility was assessed by the same sonographer via repeated measurements after an interval of 3–5 days.

### Peritonitis definitions

Peritonitis was defined according to the recent updated guidelines on PD [14]. It was diagnosed when at least two of the following factors were present: [1] clinical features consistent with peritonitis, that is, abdominal pain and/or cloudy dialysis effluent; [2] dialysis effluent white cell count > 100/µL with > 50% polymorphonuclear leukocytes; and [3] positive dialysis effluent culture (bacteria or fungi).

### Statistical analysis

Statistical analyses were made by using SPSS 27.0 software (IBM, Chicago, IL) and R software 4.1.1. Continuous variables were expressed as mean ± SD or median (25th and, 75th percentile), and categorical variables were expressed as numbers and percentages. The mean differences between groups were compared by Student’s t-tests or Mann-Whitney U test for continuous data and chi-square test for categorical data. Stepwise logistic regression analysis was employed to screen for statistically significant variables (entry *P* = 0.05, removal *P* = 0.10) and construct the regression model with peritonitis occurrence as the binary dependent variable. Then, the selected risk factors from the regression model were integrated, introducing the multivariate logistic regression analysis to construct a nomogram.

To evaluate the efficacy of individual and combined indicators in predicting diagnosis of peritonitis, we plotted the receiver operating characteristic (ROC) curve and calculated the area under the ROC curve (AUC) to evaluate the discrimination of the model. Calibration curves were drawn to evaluate the calibration of the model. Internal validation of the nomogram was performed via 1000-times bootstrap resampling. Discrimination (C-index) and calibration metrics (intercept, slope, Emax, Brier score) were calculated to quantify model stability and correct potential overfitting bias. Decision curve analysis (DCA) curve was drawn to evaluate the clinical effectiveness of the model. Validation was performed using the split-sample internal validation set randomly divided from the whole dataset. The 95% confidence intervals for sensitivity and specificity were calculated using the Wald normal approximation method. Pairwise comparisons of AUC values were performed by DeLong’s test. Statistical significance was set at *P* < 0.05.

To assess intra-observer reproducibility of ultrasonic measurements, the same experienced sonographer performed repeated measurements on the included children after an interval of 3–5 days. The intraclass correlation coefficient (ICC) was calculated, and an ICC > 0.75 was defined as good measurement agreement.

### Ethics

This study was conducted according to the principles of the Declaration of Helsinki and China’s Ethical Review Guidelines for Clinical Research Involving Children, and was approved by the Ethics committee of Tongji Hospital, Tongji Medical College, Huazhong University of Science and Technology (Approval no. TJ-IRB202508058). Due to the retrospective nature of the study, the Ethics committee of Tongji Hospital, Tongji Medical College, Huazhong University of Science and Technology waived the need of obtaining informed consent.

## Results

Eighty-two children were enrolled, and 51 peritonitis episodes were observed in 40 (49%) children. The incidence rate of peritonitis was 0.27 episodes per patient-year. The characteristics of the non-peritonitis group and peritonitis group were compared for the sex, age, weight, height, and body mass index (BMI) (Table 1) and there were no significant differences between these two groups(*p* > 0.05). The mean dialysis duration for the non-peritonitis group and peritonitis group was 7.50 (4.00, 13.00) months (range 3–20 months) and 13.00 (11.00, 15.00) months (range 4–56months), respectively, and the difference between the two groups was statistically significant (*p*<0.05). We analyzed laboratory data of the two study groups. The comparison revealed that the peritonitis group had significantly higher NLR (neutrophil-to-lymphocyte ratio) and significantly lower uric acid and albumin levels than the non-peritonitis group (*p*<0.05, Table 1). The USG measurements of the peritoneal thickness, mesenteric thickness, jejunal wall thickness and ileal wall thickness in peritonitis group were significantly thicker than the non-peritonitis group (Table 2).

Multivariable logistic regression analysis was performed to identify independent risk factors for PD-related peritonitis. After stepwise variable selection, four associated factors were retained in the final model: dialysis duration, proximal jejunal wall thickness, mesenteric thickness, and NLR. The regression coefficient (B), standard error (SE), Wald χ², P-value, odds ratio (OR), and corresponding 95% confidence interval (95%CI) of each variable were summarized in Table 3. The final multivariable logistic regression equation was as follows:

Logit(P) = − 14.401 + 0.139×Dialysis duration + 0.655×(Jejunal wall thickness×10) + 0.175×(Mesenteric thickness×10) + 0.522×NLR.

Variance inflation factors (VIF) were calculated to assess multicollinearity among dialysis duration (1.10), mesenteric thickness (1.11) and jejunal wall thickness (1.02). All VIF values were below 1.2, indicating no obvious multicollinearity, which would not affect the stability and accuracy of the multivariate Logistic regression model.

**Fig. 2:**
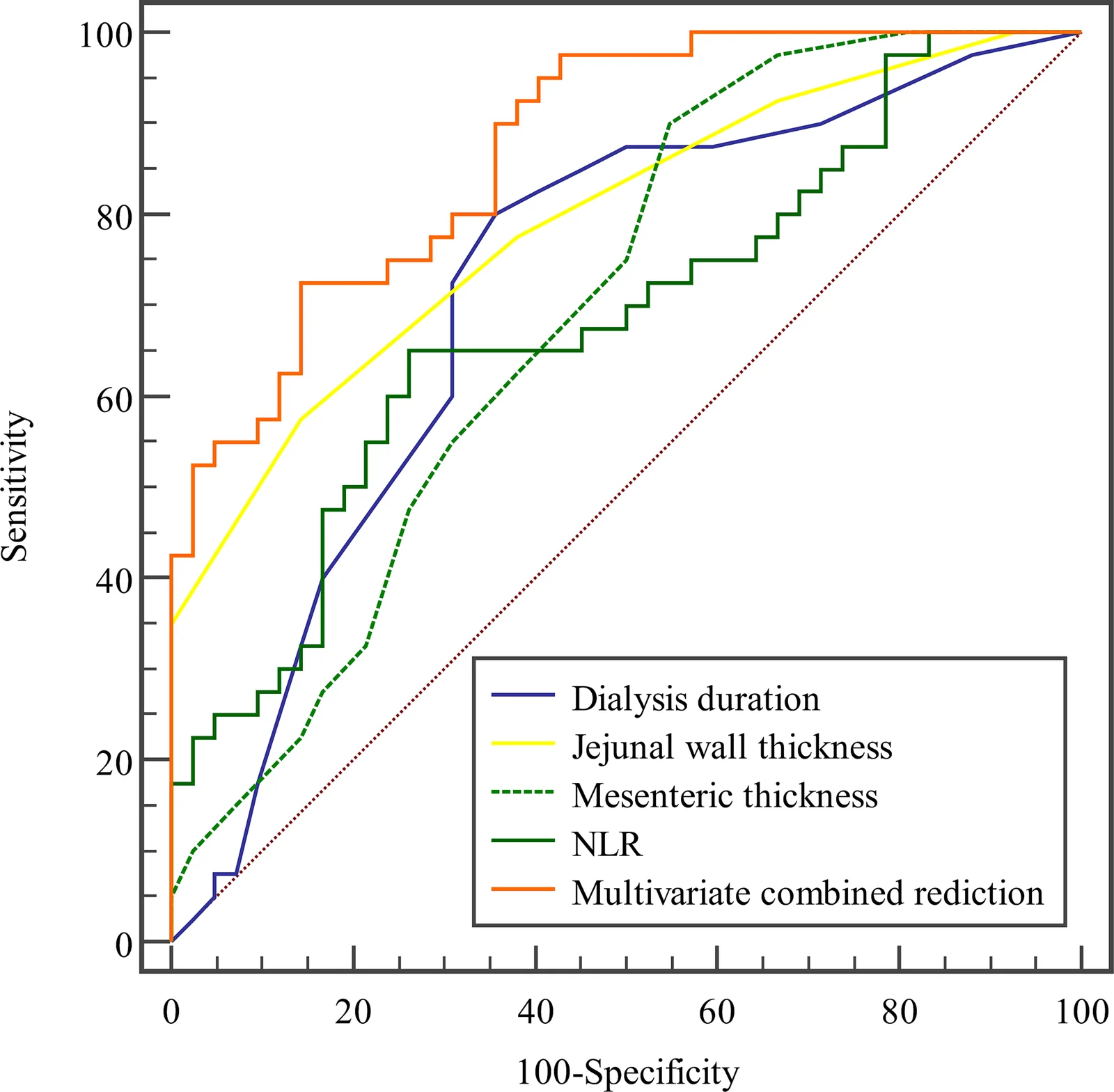
The combined prediction model yielded the highest AUC (0.873) on the ROC curve, significantly outperforming any single indicator (*P* < 0.05). ROC: Receiver operating characteristic curve. AUC: Area Under the ROC Curve

Further ROC analysis was performed to assess the diagnostic efficacy of these risk factors. The AUC for dialysis duration, mesenteric thickness, jejunal wall thickness and NLR were 0.717, 0.691, 0.793, and 0.686, respectively. The AUC of integrated predictive model was 0.873(cut-off point: 0.562), higher than any risk factors. Although the diagnostic sensitivity of dialysis duration and mesenteric thickness were 80.00% and 90.00%, their specificity were low (64.29%, 45.24%, respectively). The diagnostic sensitivity and specificity of integrated predictive model showed significantly improved, reaching 72.50% and 85.71%, respectively. DeLong’s test confirmed that the multivariate combined model had a significantly higher AUC compared with each individual predictor (all *P* < 0.05), indicating superior discriminative performance of the combined model (Table 4; Fig. 2).

**Fig. 3:**
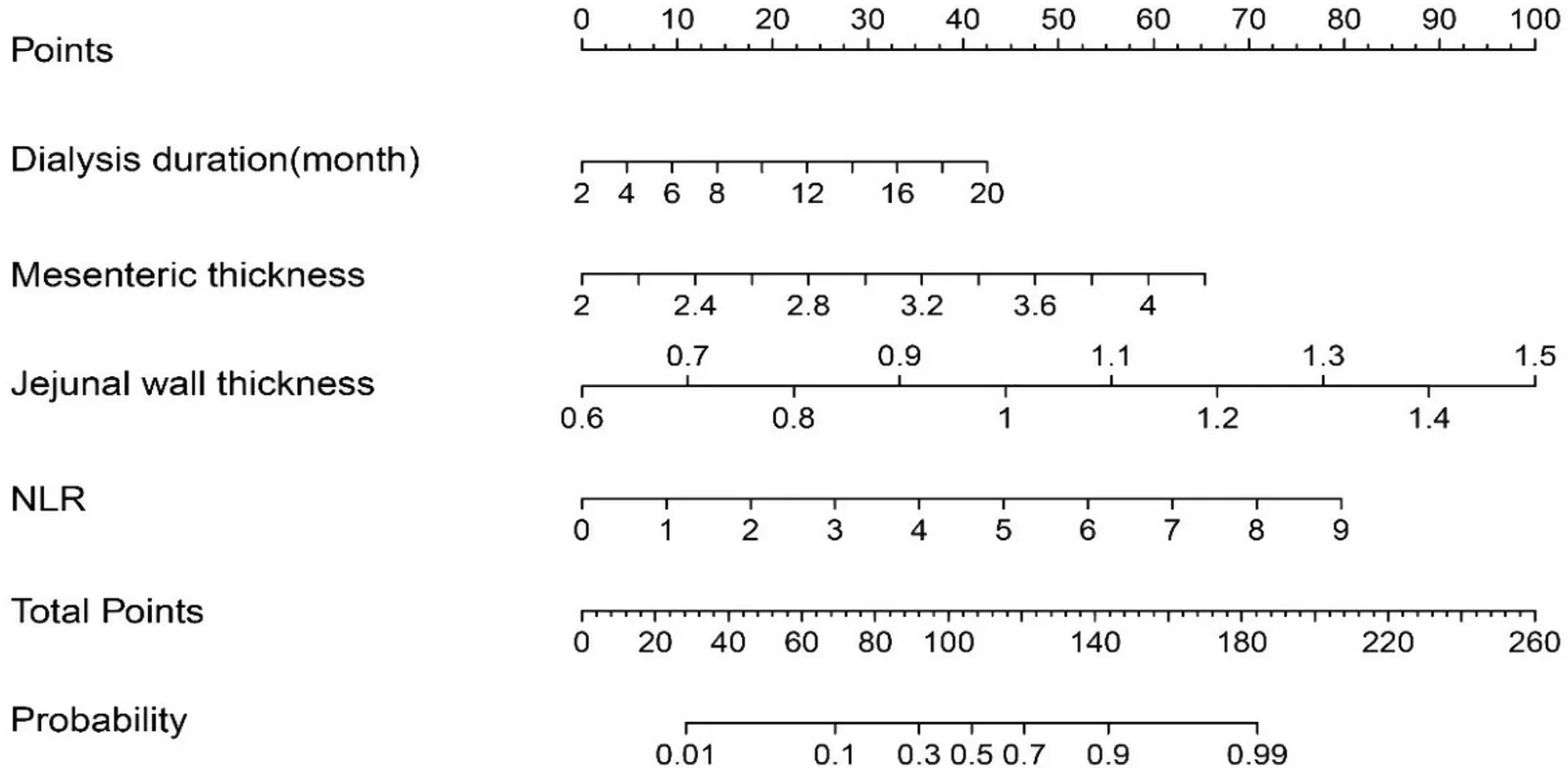
Nomogram for predicting the risk of PD-related peritonitis in children

Based on the independent risk factors identified through multivariate logistic regression analysis, we developed a visualization nomogram model (Fig. 3). Each variable’s value is represented by a numerical score, and a vertical projection is extended upward from the respective variable’s value location to intersect with the upper scoring line, thereby assigning a score to each variable. The scores of each variable are then summed up to obtain a total score. This total score is subsequently positioned along the total score reference line. From this specific point of intersection, another vertical projection is extended downward to intersect with the diagnostic probability line, ultimately obtaining the corresponding risk of developing peritonitis. The intuitive nature of the nomogram assists clinicians in rapidly assessing patient risk and making informed treatment decisions accordingly.

**Fig. 4:**
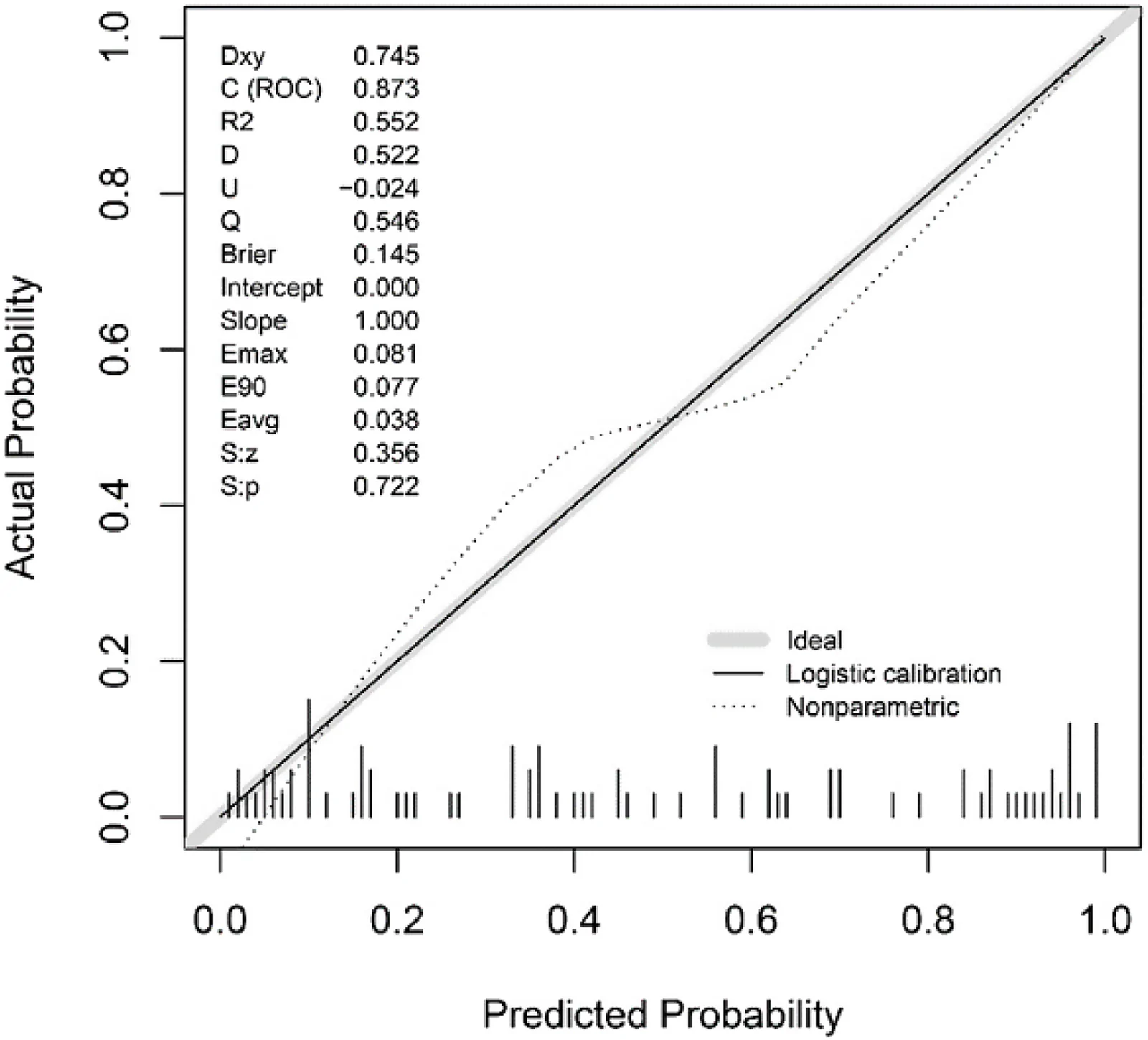
Calibration curve of the predictive nomogram after 1000-bootstrap resampling internal validation. The gray shaded line represents ideal prediction, the solid black line denotes the logistic regression-calibrated curve, and the dotted nonparametric curve reflects observed actual probability. The optimism-adjusted C-index of the model was 0.827 after eliminating overfitting bias

**Fig. 5:**
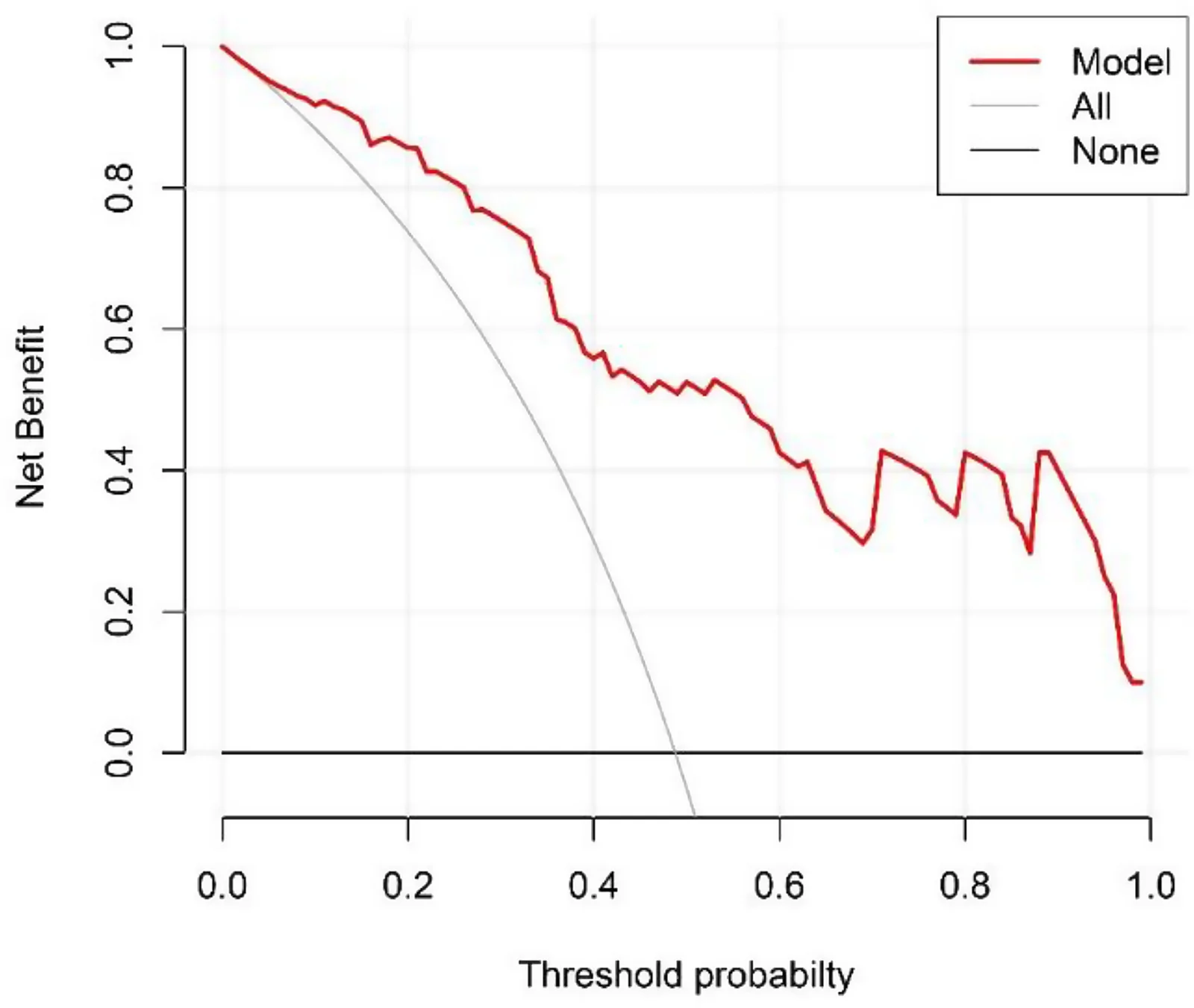
Decision curve analysis of the nomogram

The goodness-of-fit test for the regression model yielded χ² = 9.604 (*P* = 0.294), indicating satisfactory calibration performance. The calibration curve (Fig. 4) further demonstrated good agreement between the predicted and observed values. The model exhibited favorable calibration metrics: intercept = 0.000, slope = 1.000, low Emax (0.081) and Brier score (0.145). The C-index of 0.873 confirmed good discriminative performance, and these bootstrap-derived calibration results effectively verify the stability of our prediction model and reduce overfitting risk. The raw apparent C-index of the prediction model was 0.873. After 1000-times bootstrap-based internal validation per Harrell’s standard method, the shrinkage factor was 0.356 and bootstrap-corrected calibration slope was 1.000. The optimism-adjusted C-index was calculated as 0.827 after eliminating overfitting optimism bias. Meanwhile, the DCA (Fig. 5) curve demonstrates that our model maintains positive clinical net benefit across a broad threshold probability range (0 ~ 0.92), superior to the two default strategies of treating all or no patients, suggesting promising clinical utility and practical value in real-world applications.

The intra-observer ICC was 0.84 (95%CI: 0.78–0.89) for peritoneal thickness, 0.85 (95%CI: 0.80–0.90) for mesenteric thickness, 0.96 (95%CI: 0.94–0.98) for jejunal wall thickness and 0.96 (95%CI: 0.94–0.98) for ileal wall thickness, indicating good and reproducible ultrasonic measurements.

**Table 1:** Characteristics of study population

Patient characteristics	Non-peritonitis group (*n* = 42)	Peritonitis group (*n* = 40)	*p* value
Sex (male, n, %)	23(54.8)	16(40.0)	0.181
Age(years)	6.00 (4.25, 8.00)	7.00 (5.00, 9.00)	0.395
Height (cm)	111.29 ± 17.79	118.45 ± 17.10	0.067
Weight (kg)	19.50 (15.25, 23.00)	22.00 (17.75, 24.50)	0.122
BMI (kg/m2)	15.98 (14.94, 16.63)	15.80 (14.72, 16.73)	0.711
Dialysis duration	7.50 (4.00, 13.00)	13.00 (11.00, 15.00)	0.001*
Uric acid(µmol/L)	458.25 (347.75, 529.25)	358.45 (308.00, 468.50)	0.047*
Albumin(g/L)	35.10 (32.12, 38.30)	33.50 (29.95, 36.02)	0.036*
NLR	1.54 (1.08, 2.00)	2.29 (1.38, 3.43)	0.004*

*Statistically significant, BMI, Body mass index; NLR, neutrophil to lymphocyte ratio

**Table 2:** Comparison of ultrasonographic measurements between non-peritonitis group and peritonitis group

	Non-peritonitis group (*n* = 42)	Peritonitis group (*n* = 40)		*p* value
Peritoneal thickness(mm)	0.60 (0.60, 0.70)	0.70 (0.60, 0.80)	0.019	
Mesenteric thickness(mm)	2.98 ± 0.42	3.24 ± 0.35	0.002	
Jejunal wall thickness(mm)	0.80 (0.70, 0.90)	1.00 (0.90, 1.20)	< 0.001	
Ileal wall thickness(mm)	0.80 (0.70, 0.90)	0.90 (0.80, 1.02)	0.001	

**Table 3:** Multivariable logistic regression analysis for risk factors of PD-related peritonitis

Parameter	B	SE	Wald	*P*	OR	95% CI
Dialysis duration	0.139	0.062	5.000	0.025	1.149	1.017–1.299
Mesenteric thickness	0.655	0.22	8.841	0.003	1.925	1.250–2.965
Jejunal wall thickness	0.175	0.081	4.714	0.030	1.192	1.017–1.396
NLR	0.522	0.251	4.319	0.038	1.685	1.030–2.756
Const.	-14.401	3.494	16.987	< 0.001	0	

B, regression coefficient; SE, standard error; Wald, Wald chi-square value; P, P-value; OR, odds ratio; CI, confidence interval; Const., continuous variable

**Table 4:** Diagnostic performance of ultrasonographic parameters, clinical indicators and the multivariate combined model for pediatric PD-related peritonitis

Parameter	AUC	*P* value	95%CI of AUC	Cut-off point	Sensitivity (%) (95% CI)	Specificity (%) (95% CI)	*P* value (vs. combined model)
Dialysis duration	0.717	< 0.001	0.606–0.811	13	80.00(67.61–92.39)	64.29(54.90–73.68)	0.003
Mesenteric thickness	0.691	0.001	0.579–0.788	2.8	90.00(80.71–99.29)	45.24(35.48–55.00)	0.002
Jejunal wall thickness	0.793	< 0.001	0.689–0.875	0.9	57.50(42.17–72.83)	85.71(78.85–92.57)	0.022
NLR	0.686	0.002	0.574–0.784	1.936	65.00(50.22–79.78)	73.81(65.19–82.43)	0.001
Multivariate combined prediction	0.873	< 0.001	0.781–0.936	0.562	72.50(58.66–86.34)	85.71(78.85–92.57)	

AUC, area under the curve; CI, confidence interval; NLR, neutrophil-to-lymphocyte ratio. The P values (vs. combined model) were obtained from DeLong’s test comparing the AUC of each individual predictor with that of the multivariate combined prediction model

## Discussion

There have been many studies focused on the USG presence of tunnel infection or tuberculous peritonitis in adult [15–18]. However, limited USG experimental research has investigated the structural changes in the peritoneum, mesentery, and intestinal wall throughout the prolonged course of PD in pediatric populations. Different from previous studies, we performed USG measurements of parietal peritoneal, mesenteric and intestinal wall thickness across various anatomical locations in children on PD. To our knowledge this is the first report on systematic USG evaluation of the peritoneum, mesentery and intestinal wall in children with PD-related peritonitis.

Kidney failure is relatively uncommon in infants and young children, and its progression is linked to considerable morbidity and mortality. Mortality from PD–related peritonitis ranges from 3% to 10%, and the risk of death remains elevated for up to 120 days following the resolution of a peritonitis episode [19, 20]. Misaki’s research revealed that the incidence rate of peritonitis was highest among children under 2 years old [21]. The International Pediatric Peritoneal Dialysis Network registry reported an annual rate of peritonitis of 0.44 (one infection every 27 months) in children undergoing PD [9]. Our research result was 0.27(one infection every 44 months), which was slightly lower. This may be attributed to improved preventive strategies adopted in routine patient management. Alternatively, it could be attributed to an insufficient sample size.

Dialysis duration is a critical confounding factor and it differed significantly between the two groups in our study. We adjusted for dialysis duration in the regression model, but residual confounding still exists given the small sample size. After controlling for dialysis duration, the peritoneal, mesenteric and intestinal wall thickness were still correlated with peritonitis episodes. We emphasize that the observed morphological alterations are associated with both peritonitis and prolonged dialysis, and definite causal inference cannot be drawn at present.

Long-term dialysate exposure and repeated peritonitis cause peritoneal thickening via inflammation and fibrosis [11, 22, 23]. Related pathological changes include oedema, organelle disruption and collagen deposition [6, 8]. Mesothelial cells transform into myofibroblasts during fibrosis, presenting degenerative oedema [24, 25]. Ultrasound is capable of detecting peritoneal, mesenteric and intestinal thickening, along with fine fibrin strands and septations caused by recurrent peritonitis. Our findings further demonstrate that visceral peritoneal alterations correlate with peritonitis frequency, as reflected by changes in mesenteric and intestinal wall thickness.

### The parietal peritoneum

The normal peritoneal structures, including the mesenteries are composed of only a few cell layers thick and are primarily visualized in imaging studies based on their internal composition, including fatty tissue as well as vascular and lymphatic structures. These structures become more conspicuous and exhibit alterations in their radiological appearance when involved by pathological processes [26]. Williams et al. demonstrated that parietal peritoneal thickness correlated with PD duration via biopsy, and regarded peritoneal biopsy as the gold standard for assessing peritoneal morphology [6]. However, this invasive method is not suitable for routine clinical assessment, especially for children. Our findings exactly support their biopsy-specimen-based findings and consistent with other previous research [7, 11, 12, 22, 23] by means of non-invasive USG examinations.

### The mesentery

In Derchi et al. ‘s early study, it was impossible to see any portion of the mesentery only in two very thin adult patients [27]. While in our study almost every child’s mesentery can be observed by US. This reason could be attributed to advancements in technology, resulting in clearer ultrasound imaging, as well as the fact that children’s abdomens are more accessible for ultrasound examination. In normal adult subjects, the thickness of the mesentery ranges from 5 to 14 mm [27]. Q. Xia et al. measured the thickness of the normal terminal ileum mesentery in normal adult individuals and patients with mesentery-related disease. Their result of the mean thickness of normal individuals was 6.8 ± 2.4 mm, and subjects in the fatty liver group had a thicker mesentery [28]. Although adult mesenteric ultrasonography is well-described, data on pediatric cases remain scarce. We measured the mean mesenteric thickness in peritonitis group (with a mean age of 7 years) was 3.24 ± 0.35 mm, significantly thicker than the non-peritonitis group (with a mean age of 6 years), which was measured as 2.98 ± 0.42 mm. Our research data addressed this gap in the pediatric field.

### The intestinal wall

The thickness of the intestinal wall in children has been reported to range from 0.3 to 2.0 mm [29]. The thickening of the wall, as an objective and quantifiable indicator, is recognized as an accurate measurement reflecting ongoing inflammation that can be followed overtime. The intestinal wall thickness measured in our study refers to the overall thickness of the intestinal wall, including the mucosa, muscularis mucosa (not always visible or very thin), submucosa, muscularis propria, and serosa. van Wassenaer et al. reported that the normal intestinal wall thickness was 0.5–1.1 mm for the jejunum and 0.6–1.9 mm for the ileum in healthy children [30]. Our study data showed that the mean thicknesses of the proximal jejunal wall and terminal ileal wall were 1.00 mm and 0.90 mm in the peritonitis group, versus 0.80 mm and 0.80 mm in the non-peritonitis group. This could be attributed to the existence of submucosal fatty deposition or muscular hypertrophy following active inflammation [31]. Our multivariate logistic regression analysis revealed that jejunal wall thickening, rather than ileal wall thickening, was associated with peritonitis. This discrepancy may be attributed to the richer vascular supply and slightly thicker wall of the jejunum compared to the ileum. Consequently, inflammatory changes in the jejunum may manifest more prominently when pathology occurs. We also observed that intestinal wall thickening could resolve fully after effective treatment, whereas persistent thickening was seen in some children on long-term PD even without active inflammation.

### Albumin & uric acid

Serum albumin levels are typically utilized to evaluate the nutritional status of patients with CKD, and can also indicate the inflammatory status and volume status of patients undergoing PD [32]. When compared to patients with a baseline serum albumin level of ≥ 38 g/L, those with an albumin level of < 38 g/L exhibit a hazard ratio of 1.406 for the first occurrence of peritonitis [33]. Noppakun et al. found that a decrease in serum albumin before the onset of PD is an independent risk factor for peritonitis [34]. In our study, the albumin in the peritonitis group(33.5 g/L)was lower than the non-peritonitis group༈35.1 g/L༉. Thus, decreased serum albumin levels could serve as a marker of impaired immune defense, warranting vigilance for peritonitis onset in these patients.

Hyperuricemia commonly occurs in CKD patients. In our study, the uric acid in the peritonitis group(358.45 µmol/L)was lower than the non-peritonitis group༈458.25 µmol/L༉, which suggested that decreased uric acid levels were associated with an increased risk of peritonitis, potentially attributable to malnutrition or inadequate protein intake. However, the stepwise regression model did not incorporate the albumin and uric acid as risk factors, possibly owing to insufficient sample size in our study sample size. The significance of this finding remains inconclusive and require further validation.

### NLR

Neutrophil-to-lymphocyte ratio (NLR) was considered a predictor of tumor prognosis and was associated with inflammation in cancer patients initially [35]. Compared to single neutrophil or lymphocyte counts, NLR better reflected the systemic inflammatory state [36]. Several studies have suggested that NLR is negatively correlated with albumin, which is related to decreased protein synthesis, increased catabolism, and albumin translocation to the vascular compartment during inflammation [37]. Our study found that NLR level was a risk factor for peritonitis in PD patients, likely related to the aforementioned inflammatory responses and decreased albumin levels.

It is necessary to interpret mesenteric and jejunal wall thickening with caution due to multiple potential confounders. Although we ruled out patients with intestinal disease, abdominal surgery and malignancy and adopted unified protocols for dialysis and ultrasound examination to guarantee measurement reliability, supported by good intra-observer reproducibility. Hydration, nutrition and systemic inflammation remained inevitable confounders. Peritonitis patients had elevated NLR and reduced albumin and uric acid, indicating active inflammation and suboptimal nutrition. These factors may interact with repeated peritonitis and contributed to tissue thickening collectively. The morphological changes observed may be attributed to cumulative peritoneal damage, chronic inflammation and nutritional impairment, rather than peritonitis alone.

Our study aims to preliminarily develop and validate the ultrasound-based diagnostic and predictive value for recurrent peritonitis in children undergoing PD. We developed a model by converting USG measurements into quantitative features. These USG measurements in our study were obtained during stable follow-up periods at least four weeks after peritonitis resolution. These chronic morphological changes reflect cumulative peritoneal injury induced by prior inflammatory insults, and can be used to screen and stratify the long-term risk of future peritonitis recurrence. This model provides a novel, non-invasive imaging biomarker to assist clinicians in identifying high-risk pediatric PD patients before new infectious episodes occur, allowing for targeted monitoring, individualized preventive interventions, and optimized peritoneal dialysis management strategies.

Our study has several limitations. Firstly, the research was limited to the current single-center cohort with a relatively small sample size and requires external validation. Second, Stepwise regression was adopted for variable screening, which carries potential risks of model instability and overfitting. Third, as a retrospective study, this research may be subject to selection bias. Fourth, the follow-up period of this study was short, and the value of predicting recurrent peritonitis of children undergoing PD requires further validation. These results remain preliminary. Further prospective multicenter studies with larger sample sizes and more rigorous statistical methods are required before this model can be applied in routine clinical practice.

In conclusion, our study indicates that dialysis duration, mesenteric thickness, jejunal wall thickness and NLR are potential associated factors of PD-related peritonitis in children. The combined model integrating these indicators exhibits favorable predictive performance, which may provide useful ultrasonographic biomarkers for identifying high-risk patients. Collectively, these ultrasound parameters have the potential to act as imaging references to assist clinicians in optimizing dialysis management and treatment strategies.

## Data Availability

All data generated or analyzed during this study are included in this article. Further inquiries can be directed to the corresponding author.
